# Diffusion tensor imaging profiles reveal specific neural tract distortion in normal pressure hydrocephalus

**DOI:** 10.1371/journal.pone.0181624

**Published:** 2017-08-17

**Authors:** Nicole C. Keong, Alonso Pena, Stephen J. Price, Marek Czosnyka, Zofia Czosnyka, Elise E. DeVito, Charlotte R. Housden, Barbara J. Sahakian, John D. Pickard

**Affiliations:** 1 Department of Neurosurgery, National Neuroscience Institute and Duke-NUS Medical School, Singapore, Singapore; 2 Neurosurgical Division, Department of Clinical Neurosciences, University of Cambridge, Cambridge, United Kingdom; 3 SDA Bocconi School of Management, Milan, Italy; 4 Department of Psychiatry and MRC/ Wellcome Trust Behavioural and Clinical Neuroscience Institute, University of Cambridge, Cambridge, United Kingdom; 5 Department of Psychiatry, Yale University School of Medicine, New Haven, Connecticut, United States of America; Instituto Cajal-CSIC, SPAIN

## Abstract

**Background:**

The pathogenesis of normal pressure hydrocephalus (NPH) remains unclear which limits both early diagnosis and prognostication. The responsiveness to intervention of differing, complex and concurrent injury patterns on imaging have not been well-characterized. We used diffusion tensor imaging (DTI) to explore the topography and reversibility of white matter injury in NPH pre- and early after shunting.

**Methods:**

Twenty-five participants (sixteen NPH patients and nine healthy controls) underwent DTI, pre-operatively and at two weeks post-intervention in patients. We interrogated 40 datasets to generate a full panel of DTI measures and corroborated findings with plots of isotropy (p) vs. anisotropy (q).

**Results:**

Concurrent examination of DTI measures revealed distinct profiles for NPH patients vs. controls. PQ plots demonstrated that patterns of injury occupied discrete white matter districts. DTI profiles for different white matter tracts showed changes consistent with i) predominant transependymal diffusion with stretch/ compression, ii) oedema with or without stretch/ compression and iii) predominant stretch/ compression. Findings were specific to individual tracts and dependent upon their proximity to the ventricles. At two weeks post-intervention, there was a 6·7% drop in axial diffusivity (*p = 0·022)* in the posterior limb of the internal capsule, compatible with improvement in stretch/ compression, that preceded any discernible changes in clinical outcome. On PQ plots, the trajectories of the posterior limb of the internal capsule and inferior longitudinal fasciculus suggested attempted ‘round trips’. i.e. return to normality.

**Conclusion:**

DTI profiling with p:q correlation may offer a non-invasive biomarker of the characteristics of potentially reversible white matter injury.

## Introduction

Normal pressure hydrocephalus (NPH) was first described in 1965 by Hakim and Adams as “symptomatic occult hydrocephalus with ‘normal’ cerebrospinal fluid (CSF) pressures” [1]. NPH classically comprises of a triad of gait disturbance, dementia and urinary incontinence associated with ventriculomegaly in the absence of persistently elevated intraventricular CSF pressures. NPH has a prevalence of 1–2% in the elderly population. It is important to identify patients with NPH because many of its clinical features may be reversed by the insertion of a CSF shunt. However, the clinical features of NPH are commonly encountered in aging or other neurodegenerative conditions so that many patients with a potentially reversible condition are misdiagnosed as having Alzheimer's disease or vascular dementia and *vice versa* [2, 3].

The few published autopsy and biopsy studies of NPH patients have not revealed any specific neuropathological pattern for NPH. Cerebrovascular and neurodegenerative, including Alzheimer’s changes, are present in many NPH patients. Leptomeningeal fibrosis has been found but does not correlate with resistance to CSF outflow (Rcsf) [4]. The underlying pathophysiology of NPH remains unresolved, with key hypotheses suggesting structural or tissue distortion, reversal of CSF and interstitial fluid flow, failure of drainage of vasoactive metabolites, watershed ischaemia in the deep white matter, impairment of periventricular cerebral blood flow (CBF) autoregulation, and dysfunction of CSF circulation or hydrodynamics. Most NPH groups accept the likelihood that such theories of pathogenesis are not mutually exclusive and may represent different aspects of a complex ongoing process [5–7]. However, it is not clear which parts of the brain in NPH are involved with the gait disturbance, subcortical pattern of dementia and urgency of micturition.

Various MR techniques have been applied to NPH including magnetic resonance elastography (MRE) [8]. Diffusion Tensor Imaging (DTI) is able to interrogate tissue microstructure in the injured brain including traumatic brain injury and acute hydrocephalus [9–11]. Using DTI-derived mean diffusivity (MD) analysis, Ivkovic *et al*. found it possible to distinguish NPH from Alzheimer’s and Parkinson’s diseases as well as Lewy body dementia with a sensitivity of 86% and specificity of 96% [12]. However, DTI is capable of revealing more much about white matter injury than MD alone as seen, for example, post-radiotherapy and head injury [10, 13]. Using fractional anisotropy, axial and radial eigenvalues, Hattori *et al*. demonstrated alterations in corticospinal tract microstructure sufficient to discriminate NPH from Alzheimer’s and Parkinson’s diseases, with a sensitivity of 94% and specificity of 80% [14]. Early work has demonstrated that such analyses may be used to characterize the topography and possible mechanism of injury to the white matter in NPH [15].

We hypothesized that, in NPH, differing patterns of white matter injury could be demonstrated to occur concurrently. Our study aimed to demonstrate the effects of surgical intervention on pre-operative patterns of injury and to evaluate the responsiveness of individual DTI patterns to CSF shunting. We used a full panel of DTI measures to interrogate both the microstructural effects of NPH on key white matter tracts likely to be ‘at-risk’ of damage. Published guidelines recommend the earliest assessment of clinical outcome scoring to be at 3-months post-intervention [16]. We examined patients at two weeks post-shunting, prior to expected clinically discernible changes in improvement, in order to examine the pure effect of surgical intervention on diffusion tensor imaging profiles.

## Materials and methods

### Participants

We recruited sixteen patients with NPH (ten males and six females) ranging in age from 60 to 84 years (mean age of 75 years; Table 1 for clinical characteristics of patients). The control group comprised of nine age-matched healthy controls (four males, five females; mean age of 70 years). The local Research Ethics Committee of the Cambridge Health Authority approved this study (LREC: 06/Q0108/330). Informed written consent was obtained from the patients and from the healthy controls, according to the Declaration of Helsinki. Informed written assent was obtained from patients’ family members in cases of significant cognitive impairment. All patients were assessed in a multidisciplinary cerebrospinal fluid (CSF) disorders clinic according to published NPH guidelines [17]. Ventriculomegaly was assessed using the bicaudate index (BCI); this is derived as the minimum intercaudate distance divided by the brain width along the same line. Significant ventriculomegaly is defined as a ratio of ≥ 0.25 using this method [18]. Supplementary testing included computerized CSF infusion studies (see Guidelines on supplemental testing in NPH [19]; our experience since 1992 is that an Rcsf of ≥ 13 mmHg/ml/min is significantly correlated to a favourable response following surgery, i.e. improvement in at least one clinical symptom [20]. Patients were assessed pre-operatively and at two weeks post-intervention (one patient did not attend post-operatively).

**Table 1:** Clinical characteristics of patients.

Pt	Age	Sex	BCI	Radiological abnormalities	NPH symptoms	Investigations
1	69	M	0.26	Hydrocephalus and PVL, atrophy, DWMH	Gait, memory, urinary incontinence	Rcsf = 20.80 mmHg/ml/min
2	78	F	0.26	Hydrocephalus and PVL, no atrophy or DWMH	Predominant memory disturbance	Rcsf = 16.37 mmHg/ml/min
3	70	F	0.40	Hydrocephalus and PVL, DWMH	Gait and balance disturbance, memory impairment, urinary incontinence	Rcsf = 12.40 mmHg/ml/min
4	76	M	0.26	Hydrocephalus and PVL	Gait and balance disturbance, memory impairment, urinary incontinence	Lumbar drainage positive
5	78	M	0.28	Hydrocephalus and PVL, DWMH	Gait and balance disturbance, mild memory impairment	Lumbar drainage positive, Rcsf via Ommaya reservoir = 10.51mmHg/ml/min
6	72	M	0.34	Hydrocephalus and PVL	Gait and balance problems, memory impairment	Rcsf = 13.91 mmHg/ml/min
7	60	M	0.30	Hydrocephalus and PVL	Predominant gait and balance problems, mild memory impairment, urinary frequency	Rcsf = 12.94 mmHg/ml/min
8	78	M	0.26	Hydrocephalus and PVL, atrophy, enlarged peri-Sylvian spaces, DWMH	Predominant gait and balance problems, mild memory impairment	Lumbar drainage positive
9	70	F	0.29	Hydrocephalus and PVL, No atrophy or DWMH	Predominant gait and balance disturbance, mild memory impairment	Rcsf = 20.02 mmHg/ml/min
10	78	M	0.27	Hydrocephalus and PVL, atrophy and DWMH	Gait and balance problems, memory impairment, urinary incontinence	Rcsf = 13.80 mmHg/ml/min
11	82	M	0.28	Hydrocephalus and PVL, DWMH	Gait and balance problems, memory disturbance and urinary incontinence	Rcsf = 14.41 mmHg/ml/min
12	71	M	0.32	Hydrocephalus and PVL, no atrophy or DWMH	Gait and balance disturbance, urinary frequency, memory impairment	Lumbar drainage positive
13	84	F	0.26	Hydrocephalus and PVL, atrophy, enlarged peri-Sylvian spaces, DWMH	Predominant gait and balance disturbance, mild memory impairment, nocturia	Rcsf = 13.95 mmHg/ml/min
14	74	F	0.32	Hydrocephalus and PVL, DWMH	Gait and balance disturbance, memory impairment, urinary incontinence	Rcsf = 10.92 mmHg/ml/min
15	76	M	0.26	Hydrocephalus and PVL, DWMH and enlarged peri-Sylvian spaces	Gait and balance disturbance, memory impairment, urinary incontinence	Rcsf = 24.41 mmHg/ml/min
16	79	F	0.31	Hydrocephalus and PVL, DWMH, enlarged peri- Sylvian spaces	Predominant gait and balance disturbance, memory impairment	Rcsf = 11.00 mmHg/ml/min

BCI = bicaudate index, DWMH = deep white matter hyperintensities, Pt = patient, PVL = periventricular lucency, Rcsf = resistance to cerebrospinal fluid outflow

### Neuropsychological assessment

Patients were screened pre-operatively using neuropsychological assessments to assess mood and estimated pre-morbid IQ. The following measures were used as assessments of global function (Mini Mental State Examination (MMSE), learning and memory (Hopkins Verbal Learning Task-Revised (HVLT-R), verbal fluency and executive function (COWAT) [21–23]. As expected, cognitive impairment was demonstrated in the NPH group with means for MMSE, HVLT-R and COWAT of 24.13 (dementing range ≤ 23), 3.50 and 10.21 vs. the control group means of 28.57, 8.00 and 21.29 respectively [24, 25].

### MRI studies

MRI was performed on a 3T Siemens Tim Trio using a 12 channel head matrix radio frequency receive coil. We performed imaging pre-operatively and at two weeks post-operatively. Healthy controls underwent one MR scan. The MR imaging protocol included a T_2_-weighted sequence (fast spin echo pulse sequence was acquired with FOV 224mmx168mm, 5mm slice separation, giving a voxel size 0.7x0.7x5mm; TR = 4600ms, TE = 104ms), a fluid attenuated inversion recovery (FLAIR) sequence, (TR = 7840, TE = 95ms with an inversion time T1 = 2500ms), a 3D volumetric T_1_-weighted (MPRAGE) acquisition (TR = 2300, TE = 2.98ms, with a resolution of 1x1x1mm). The DTI data set was acquired by using a spin echo diffusion-weighted echo planar imaging sequence with the following parameters: TR/TE, 8300ms/98ms, matrix dimensions 96x96, FOV 192x192, slice thickness 2mm giving a voxel size of 2x2x2mm. Diffusion weighted images were acquired in 12 non collinear directions each at 5 b-values of 350, 650, 1000, 1300 and 1600 s/mm^2^, along with 4 b-0 images.

### DTI post-processing

We analysed forty DTI datasets. We applied eddy current correction and DTI pre- and post-processing in the standard manner using the FDT diffusion toolbox in FSL analysis tools (FMRIB, Oxford, UK) [26–29]. We generated a full panel of DTI measures for each patient—fractional anisotropy (FA), mean diffusivity (MD) as well as Eigenvalues *λ*_1_, *λ*_2_ and *λ*_3_. λ_1_ was taken to represent diffusivity along the axonal fibres, i.e. axial diffusivity (λ_║_ or L1). The means of diffusivities perpendicular to the axonal fibres, *λ*_2_ and *λ*_3,_ represented radial diffusivity (λ_┴_ or L2and3). Co-registration of the structural 3D volumetric image and DTI images was performed using SPM5 (University College London, London, UK) [30]. We re-sliced the MPRAGE image to match the DTI image space using the s_0_ volume (the b = 0 volume with optimal signal-to-noise ratio).

### Generation of regions-of-interest (ROIs) using the co-registered data sets

**Fig 1:**
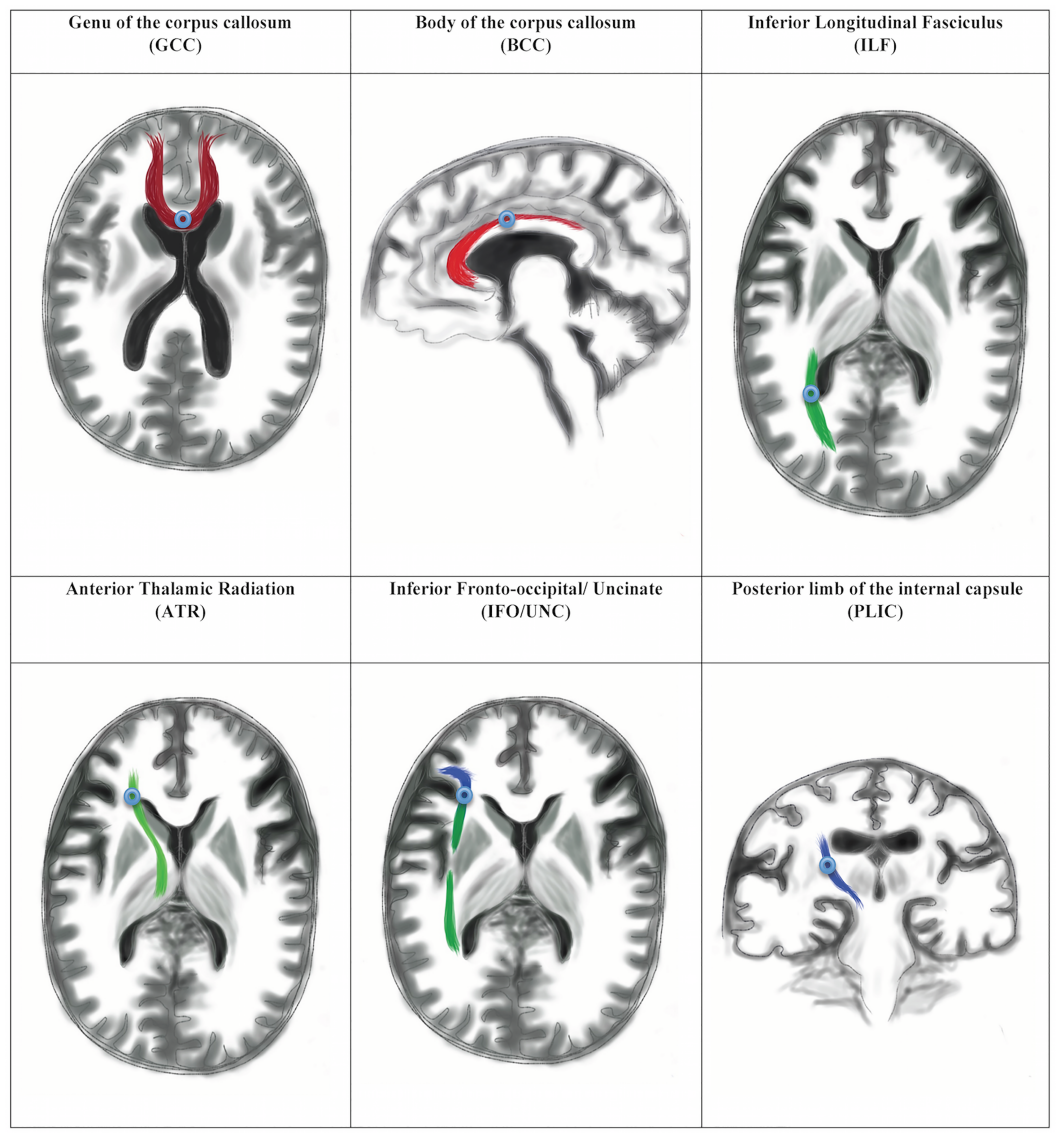
Illustration of white matter regions of interest (ROIs). White matter tracts represented in the context of normal ventricular size for clarity.

Co-registration and reproducibility of the MarsBaR methodology from the SPM5 toolbox have been previously published from our unit [10]. Prior to the study, we performed pilot work using seventy-four white matter ROIs to refine the technique for NPH, accounting for significant ventriculomegaly and surgical considerations. We used a DTI-based white matter tract atlas for anatomical identification of key fibre bundles on the structural 3D volumetric image [31]. Exclusions were performed for a variety of reasons, including relevance of tracts to NPH pathophysiology, technical consistency of ROI results, and proximity or impact from shunt/valve artefacts on post-operative imaging (Table 2). We refined the pilot ROIs into an ‘at-risk’ model of white matter injury in NPH comprising six white matter tracts (Fig 1)—the genu and body of the corpus callosum (GCC and BCC), inferior longitudinal fasciculus (ILF), anterior thalamic radiation (ATR), the junction of the inferior fronto-occipital/ uncinate fasciculi (IFO/UNC) and posterior limb of the internal capsule (PLIC). The final ROIs chosen demonstrated no significant differences in DTI parameters due to shunt/valve artefact on pilot testing. These six ROIs were also deemed highly relevant to NPH neuroanatomy. We found a 113 mm^3^ spherical MarsBaR (3mm radius) to be the optimal ROI with maximal white matter and minimal CSF/ extra-axonal sampling. We believe such a refined target avoids the limitations associated with spatial normalization.

**Table 2:** Refinement of white matter tracts into an ‘at-risk’ model of injury in NPH.

ROI	Grouping	Relevance	Discussion
SLF	Cognitive	Excluded	Whilst spatial disorientation, for example, topographical deficits, is a feature of the NPH spectrum, language disorders are not typical. This tract, with its long anterior-posterior arrangement was initially explored but found to be too prone to valve artefact.
	Spatial/ language		
ILF	Cognitive	Included	Cognitive dysfunction, such as inability to match visual recognition of common objects with the functional sequences required to use them, is a feature of the NPH spectrum. This tract was included for its relevance; its long anterior-posterior arrangement adjacent to the ventricles. Its inferior position in relation to the SLF protected this tract from issues related to valve artefact.
	Visual recognition	P = lateral	
IFO	Cognitive	Included	Whilst language disorders are not typical of the NPH spectrum, the long anterior-posterior arrangement of this tract and its fronto-basal position provided relevance to the frontal executive dysfunction seen in NPH. This tract was included but only specifically at the fronto-temporal point, i.e. the area of most likely distortion due to expansion of the frontal horns (combined with the UNC).
	Language	P = relatively remote	
UNC	Cognitive	Included	The fronto-temporal arrangement of this tract and its participation in the limbic pathway provided relevance to the frontal executive dysfunction seen in NPH. This tract was included at the area of most likely distortion due to expansion of the frontal horns, i.e. at its point of curvature. Due to considerations of crossing fibres and the relatively reduced white matter fibre bundle sizes seen in NPH, this ROI was combined with the adjacent IFO tract at this point for purposes of enhanced consistency.
	Ventral limbic	P = relatively remote	
CING	Cognitive	Excluded	The relationship of this tract to the ventricles and its participation in the limbic pathway provided relevance to the frontal executive dysfunction seen in NPH. However, the relatively large amount of surrounding CSF represented in the ROI for the cingulum bundle rendered the interpretation of this tract difficult using this methodology, and resulted in its exclusion.
	Dorsal limbic		
GCC	Motor	Included	The close relationship with this tract to the ventricles and its participation in the motor pathway provided a clear relevance to the gait and balance apraxia seen in NPH. This part of the corpus callosum was included for its anterior/ frontal location.
	Contralateral executive	P = adjacent	
BCC	Motor	Included	The close relationship with this tract to the ventricles and its participation in the motor pathway provided a clear relevance to the gait and balance apraxia seen in NPH. This part of the corpus callosum was included for its midpoint location between the two ventricles and its role in a potential hydrocephalic disconnection syndrome.
	Contralateral executive	P = adjacent	
SCC	Motor	Excluded	The close relationship with this tract to the ventricles and its participation in the motor pathway provided a clear relevance to the gait and balance apraxia seen in NPH. However, the GCC and BCC were deemed to be the stronger and more relevant ROIs.
	Contralateral executive		
ALIC	Cognitive	Excluded	Behavioural syndromes are possible in, but not typical of, NPH. The PLIC tract was also deemed to be far superior as a ROI in both functional and neuroanatomical considerations.
	Behaviour		
ATR	Cognitive	Included	This thalamocortical fibre pathway is found in the medial portion of the ALIC. However, its prefrontal cortical projections provide more relevance than ALIC to the likely areas of distortion due to the expansion of the frontal horns. This tract has been implicated both in poor cognitive performance in vascular dementia and psychopathology, such as schizophrenia, and was therefore included.
	Behaviour	P = lateral	
GIC	Sensory	Excluded	Sensory syndromes are not typical of NPH. The PLIC tract was deemed far superior as a ROI.
PLIC	Motor	Included	The key role of the corticospinal tract in gait and balance dysfunction provided a clear relevance to NPH. In addition, the superior-inferior arrangement of this tract, relatively remote to the ventricles, provided a good comparator to other relevant tracts that were adjacent and lateral to the ventricles.
		P = relatively remote	

SLF = superior longitudinal fasciculus, ILF, inferior longitudinal fasciculus, IFO = inferior fronto-occipital fasciculus, UNC = uncinate fasciculus, CING = cingulum, GCC = genu of the corpus callosum, BCC = body of the corpus callosum, SCC = splenium of the corpus callosum, ALIC = anterior limb of the internal capsule, GIC = genu of the internal capsule, PLIC = posterior limb of the internal capsule, P = position in relation to lateral ventricles

ROIs were exported to Image J (National Institutes of Health, Bethesda, USA) and applied to all DTI output files [32]. We visually inspected ROI placements on each DTI output file for quality of co-registration. Right and left ROIs were analyzed separately and the means calculated for each ROI; no significant differences were found between right and left on pilot data. The same operator (N.K.) generated all ROIs for consistency. Reliability measures were performed using ROI methodology on a training dataset of ten patients with NPH. Intraclass correlation coefficients (ICCs) generated using FA and MD measures for the GCC, BCC and PLIC ranged from 0.811 to 0.998 (intra-rater) and 0.802 to 0.995 (inter-rater) with the use of strict pre-determined neuroanatomical landmarks. We further tested the training dataset for reliability against an independent graphical software toolbox to account for variability in techniques described in the literature [33]. ICCs generated using FA and MD measures for GCC and BCC ROIs ranged from 0.941 to 0.995 using ImageJ vs. ExploreDTI [34], suggesting that results from ROI methodology were reproducible across different preferred software tools.

### Statistical analysis and DTI profiles

Descriptive statistical analysis was performed using the SPSS 15.0 (SPSS Software Products, Chicago, USA). Independent-samples *t*-tests (two-tailed) were performed for patients compared to controls and paired-samples *t*-tests (two-sided) for pre-operative compared to post-operative patients. Results were deemed to be significant at a value of *p < 0*.*05* on the Student’s *t*-test. We considered each white matter tract independently of each other; only two groups were compared in each experiment. Multivariate ANOVA-type analyses of DTI measures were not performed; ROI sets for each pairing were directly compared for the analysis of directional information and proportional magnitude of changes in order to generate DTI profiles; Bonferroni corrections were therefore not deemed appropriate in this study. We performed comparison of means and the percentage differences of changes in fractional anisotropy as well as mean, axial and radial diffusivities. By correlating significant findings with the simultaneous examination of the direction and magnitude of changes across the full panel of DTI measures, we determined predominant patterns of injury. Interpretation of DTI measures was determined *a priori* and derived from work published in human and animal studies (see Discussion). DTI patterns were further interrogated using published p:q tensor decomposition methodology for consistency [35].

### The p:q tensor transformation

We have previously introduced p:q tensor transformation for the analysis of MR DTI images in Pena *et al* [35]. This technique is based on the classical decomposition of the diffusion tensor into its isotropic and anisotropic components, as already observed by Basser and Pierpaoli *et al* [28, 36]. Our contribution was to construct a graphical representation of the diffusion tensor based on this decomposition that would facilitate the analysis and visualization of large datasets of region of interest (ROI) and voxel-based (VB) imaging data. The p:q tensor decomposition enhances the visualization and quantification of MR DTI data in both normal and pathological conditions. In particular, it is an aid to simultaneously visualize seven scalar tensor measures; we showed this in the case of a normal volunteer, a stroke patient and a hydrocephalus patient.

The first step in the p:q tensor transformation is to decompose the diffusion tensor D into two tensors P and Q according to the next equation:

$$D_{ij}\;=\;\underset{P_{ij}}{\underset{︸}{D\;I_{ij}}}\;+\;\underset{Q_{ij}}{\underset{︸}{\left[D_{ij}\;-\;D\;I_{ij}\right]}}$$

Here I is the identity tensor

$$I_{ij}\;=\;diag\left(1,\;1,\;1\right)$$

The first term on the right hand side is the isotropic tensor (denoted tensor P), while the second term in brackets represents the deviatoric tensor (denoted tensor Q). The magnitude of these tensors can be quantified by its scalar isotropic (p) and anisotropic (q) components. The values of p and q can be computed as:

$$p\;=\;\sqrt{3}\;D$$

$$q\;=\;\sqrt{{\left(\lambda_{1}\;-\;D\right)}^{2}\;+\;{\left(\lambda_{2}\;-\;D\right)}^{2}\;+\;{\left(\lambda_{3}\;-\;D\right)}^{2}}$$

Here D is the mean diffusion and the lambdas are the eigenvalues of the diffusion tensor. According to these definitions, p is therefore a scaled measure of the mean diffusion in the tensor, while q is a measure of the variance or deviation of the eigenvalues with respect to the mean diffusion of the tensor.

The second step is to plot each tensor as a point in a Cartesian plane with p taken as the x-axis and q as the y-axis. This plane will be denoted as the p:q plane. The effect of this transformation is to reduce the dimensionality of the tensor from six dimensions to two.

The third step is to use the p:q plane to deduce the five additional tensor scalar measures: D, RA, FA, L and phi. Four of these seven tensor measures (q, RA, FA, phi) are anisotropy measures, while D and p are measures of the magnitude of diffusion and L is a measure of the total diffusion of the tensor. D, p, q and L have units of x10^-4^ mm^2^/s, RA and FA are dimensionless, and phi has units of degrees.

These scalar measures can be deduced either analytically or graphically. The analytical method is to directly compute the measures based on the p, q components using the formulae:

$$\begin{array}{l} L\;=\;\sqrt{p^{2}\;+\;q^{2}}\\ RA\;=\;\frac{q}{p}\\ FA\;=\;\sqrt{\frac{3}{2}}\;\frac{q}{L}\\ phi\;=\;{\text{tan}}^{-1}\;\left(\frac{q}{p}\right) \end{array}$$

In previous work, we highlighted the potential pitfalls of using FA and RA as sole means of MR DTI analysis and the advantage of visually representing seven scalar measures simultaneously. The p:q tensor transformation has since been used in various clinical settings to quantify the diffusion and anisotropy in various tissues, thereby gaining information about their internal architecture. Srinivasan *et al*. have recently used it to distinguish intracranial epidermoids from normal white matter in the splenium of the corpus callosum [37]. In this study, we first examined concurrent changes in both magnitude and direction across the full panel of DTI measures, and interpreted them; we further corroborated this graphically by using PQ plots. In addition, we tested the interpretation of trends by comparing changes seen to mechanical stretch curves, as per Ateshian and Weiss [38], which generated predictions for theoretical directions of change in the context of decompression.

### Role of the funding source

The sponsors of the study had no role in study design, data collection, data analysis, data interpretation, or writing of the report. All authors had full access to all the data in the study and responsibility for the decision to submit for publication.

## Results

### Imaging analysis

#### Pre-operative NPH patients vs. healthy age-matched controls (Tables 3 and 4)

In NPH patients compared to controls, there was an increase in MD in all tracts with the greatest change in the corpus callosum (genu +33.9%, *p = 0*.*001*; and body +26.1%, *p = 0*.*002*; Table 3). This large increase in MD in the corpus callosum was accompanied by a decrease in FA (-21.6%, *p = 0*.*001* and -21.9%, *p = 0*.*003* respectively; Table 3) due to a large increase in radial diffusivity, L2and3, (+92.7%, *p = 0*.*002* and +48.3%, *p = 0*.*002*; Table 4) despite smaller but significant increases in axial diffusivity, L1, (+21.9, *p = 0*.*017* and +10.4%, *p = 0*.*029*; Table 4). All these changes were significant.

**Table 3:** Pre-operative vs. early post-operative NPH patients and Healthy controls (HC)–mean (SD), *t*-tests: FA and MD measures.

DTI measure	Status	GCC	BCC	ILF	ATR	IFO/UNC	PLIC
**FA**	**Pre-op patients**	0.602 (0.128)	0.478 (0.097)	0.545 (0.051)	0.380 (0.060)	0.403 (0.059)	0.751 (0.034)
	**Post-op patients**	0.571 (0.143)	0.470 (0.071)	0.519 (0.069)	0.383 (0.045)	0.421 (0.068)	0.682 (0.066)
	**Healthy controls (HC)**	0.768 (0.023)	0.612 (0.090)	0.575 (0.070)	0.452 (0.046)	0.399 (0.048)	0.713 (0.064)
	**Pre-op vs. HC (% difference)**	-21.6	-21.9	-5.2	-15.9	+1.0	+5.3
(x10^-4^mm^2^/s)	**(p value)**	(***p = 0*.*001)***	(***p = 0*.*003)***	(*p = 0*.*231)*	(***p = 0*.*005)***	(*p = 0*.*863)*	(*p = 0*.*064)*
	**Post-op vs. Pre-op (% difference)**	-5.1	-1.7	-4.8	+0.8	+4.5	-9.2
	**(p value)**	(*p = 0*.*519)*	*(p = 0*.*798)*	*(p = 0*.*245)*	*(p = 0*.*887)*	*(p = 0*.*432)*	***(p = 0*.*001)***
	**Post-op vs. HC (% difference)**	-25.7	-23.2	-9.7	-15.3	+5.5	-4.3
	**(p value)**	***(p = 0*.*001)***	***(p = 0*.*000)***	*(p = 0*.*071)*	***(p = 0*.*001)***	*(p = 0*.*395)*	*(p = 0*.*280)*
**MD**	**Pre-op patients**	8.295 (1.569)	9.348 (1.454)	7.807 (0.692)	7.045 (0.360)	7.140 (0.421)	5.879 (0.440)
	**Post-op patients**	8.226 (1.847)	10.347 (1.353)	7.662 (0.586)	7.220 (0.939)	7.000 (0.393)	5.899 (0.423)
	**Healthy controls (HC)**	6.196 (0.341)	7.416 (1.078)	6.610 (1.143)	6.633 (0.558)	6.550 (0.380)	5.350 (0.299)
	**Pre-op vs. HC (% difference)**	+33.9	+26.1	+18.1	+6.2	+9.0	+9.9
(x10^-4^mm^2^/s)	**(p value)**	***(p = 0*.*001)***	(***p = 0*.*002)***	(***p = 0*.*003)***	(***p = 0*.*034)***	(***p = 0*.*002)***	(***p = 0*.*004)***
	**Post-op vs. Pre-op (% difference)**	-0.8	+10.7	-1.9	+2.5	-2.0	+0.3
	**(p value)**	*(p = 0*.*911)*	*(p = 0*.*058)*	*(p = 0*.*536)*	*(p = 0*.*493)*	*(p = 0*.*348)*	*(p = 0*.*897)*
	**Post-op vs. HC (% difference)**	+32.8	+39.5	+15.9	+8.8	+6.9	+10.3
	**(p value)**	***(p = 0*.*004)***	***(p = 0*.*000)***	***(p = 0*.*007)***	*(p = 0*.*104)*	***(p = 0*.*012)***	***(p = 0*.*003)***

Shading in grey illustrates differences in white matter injury patterns were significant.

**Table 4:** Pre-operative vs. early post-operative NPH patients and Healthy controls (HC)–mean (SD), *t*-tests: Axial, radial diffusivities.

DTI measure	Status	GCC	BCC	ILF	ATR	IFO/UNC	PLIC
**L1**	**Pre-op patients**	14.570 (1.058)	14.430 (1.503)	13.040 (1.377)	10.042 (0.566)	10.430 (0.789)	12.377 (1.011)
	**Post-op patients**	14.110 (2.134)	15.548 (1.421)	12.512 (1.140)	10.290 (1.209)	10.375 (0.767)	11.550 (0.870)
	**Healthy controls**	11.949 (3.875)	13.072 (1.191)	11.302 (1.688)	10.214 (0.909)	9.499 (0.569)	9.677 (3.398)
	**Pre-op vs. HC (% difference)**	+21.9	+10.4	+15.4	-1.7	+9.8	+27.9
(x10^-4^mm^2^/s)	**(p value)**	(***p = 0*.*017)***	(***p = 0*.*029)***	***(p = 0*.*010)***	*(p = 0*.*564)*	(***p = 0*.*005)***	***(p = 0*.*006)***
	**Post-op vs. Pre-op (% difference)**	-3.2	+7.7	-4.0	+2.5	-0.5	-6.7
	**(p value)**	*(p = 0*.*431)*	***(p = 0*.*027)***	*(p = 0*.*253)*	*(p = 0*.*474)*	*(p = 0*.*929)*	***(p = 0*.*022)***
	**Post-op vs. HC (% difference)**	+18.1	+18.9	+10.7	+0.7	+9.2	+19.4
	**(p value)**	*(p = 0*.*090)*	***(p = 0*.*000)***	***(p = 0*.*047)***	*(p = 0*.*871)*	***(p = 0*.*008)***	*(p = 0*.*052)*
**L2and3**	**Pre-op patients**	5.158 (2.047)	6.807 (1.671)	5.191 (0.547)	5.547 (0.500)	5.358 (0.738)	3.255 (2.013)
	**Post-op patients**	5.530 (2.113)	8.363 (3.143)	5.643 (1.893)	6.071 (1.758)	5.744 (2.033)	4.395 (2.923)
	**Healthy controls (HC)**	2.677 (0.239)	4.589 (1.291)	4.264 (0.949)	4.843 (0.497)	5.075 (0.425)	2.621 (0.386)
	**Pre-op vs. HC (% difference)**	+92.7	+48.3	+21.7	+14.5	+5.6	+24.2
(x10^-4^mm^2^/s)	**(p value)**	***(p = 0*.*002)***	***(p = 0*.*002)***	(***p = 0*.*005)***	(***p = 0*.*003)***	(*p = 0*.*306)*	(*p = 0*.*392)*
	**Post-op vs. Pre-op (% difference)**	+7.2	+22.9	+8.7	+9.4	+7.2	+35.0
	**(p value)**	*(p = 0*.*622)*	*(p = 0*.*093)*	*(p = 0*.*367)*	*(p = 0*.*262)*	*(p = 0*.*482)*	*(p = 0*.*213)*
	**Post-op vs. HC (% difference)**	+106.6	+82.2	+32.3	+25.4	+13.2	+67.7
	**(p value)**	***(p = 0*.*001)***	***(p = 0*.*003)***	*(p = 0*.*055)*	*(p = 0*.*055)*	*(p = 0*.*345)*	*(p = 0*.*106)*

Shading in grey indicates that differences in white matter injury patterns were significant.

The inferior longitudinal fasciculus, anterior thalamic radiation and inferior fronto-occipital/ uncinate fasciculi demonstrated similar but less marked changes. In contrast, the increase in axial diffusivity in the posterior limb of the internal capsule was sufficiently large (+27.9%; *p = 0*.*006*) to result in a small increase, not a decrease, in FA (+5.3%; p = 0.064) despite an increase in radial diffusivity (+24.2%; p = 0·392).

**Fig 2:**
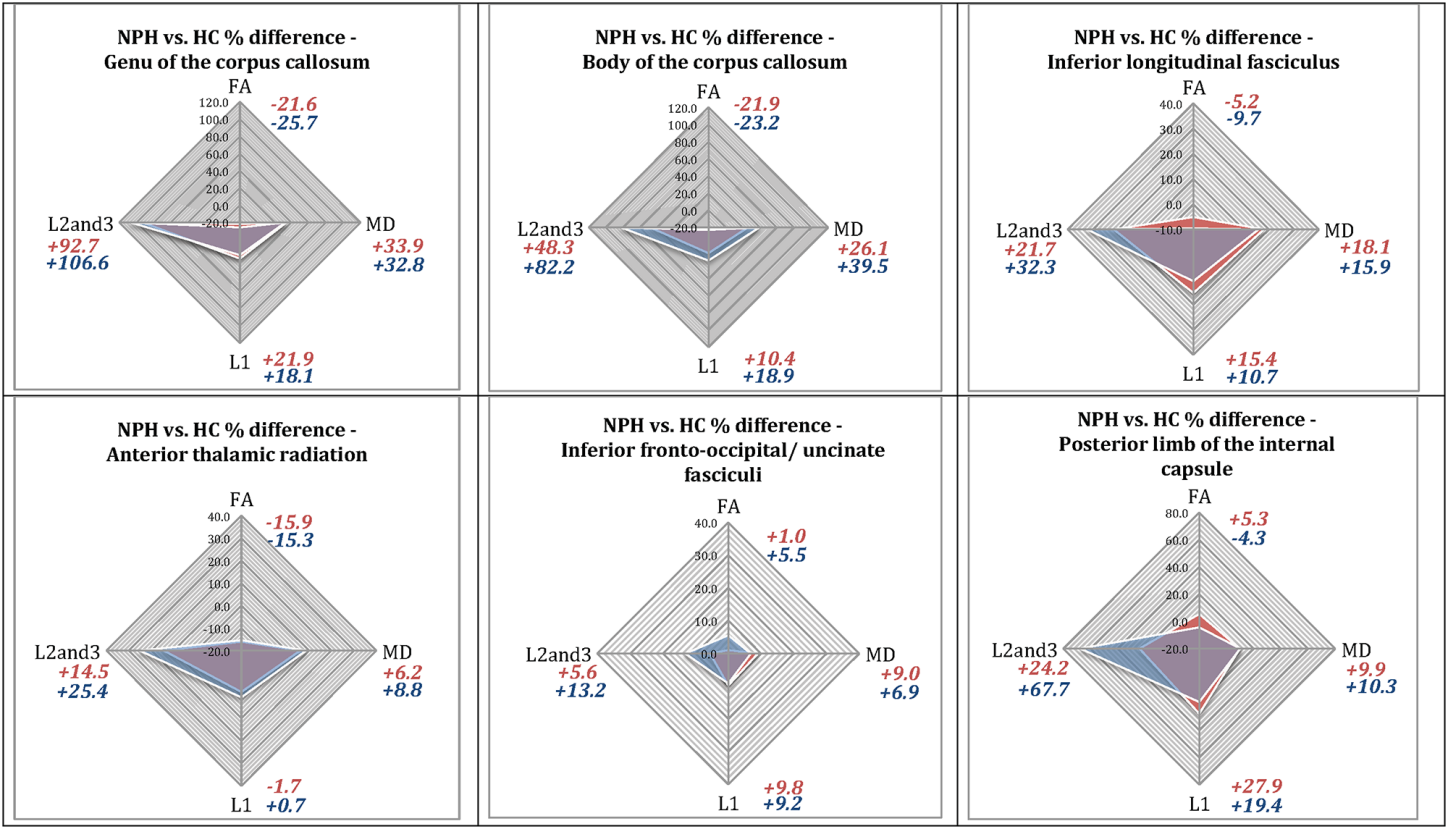
DTI profiles in pre-operative and post-operative NPH patients vs. healthy controls (in percentage difference (%)). Illustration of DTI profiles—Four measures comprising the full panel for DTI interpretation, (FA, MD, L1 and L2and3), are visually represented as radar graphs. Differences in measures may be viewed concurrently; each axis is scalar and dimensionally comparable. The morphology of the graph, i.e. the shape of the radar web, is dependent upon i) the degree of differences between the two groups compared (the larger the differences, the bigger the area of the radar web) and ii) the DTI measure predominantly affected (the radar web points towards the DTI measure with the highest percentage difference between the groups).

**Fig 3:**
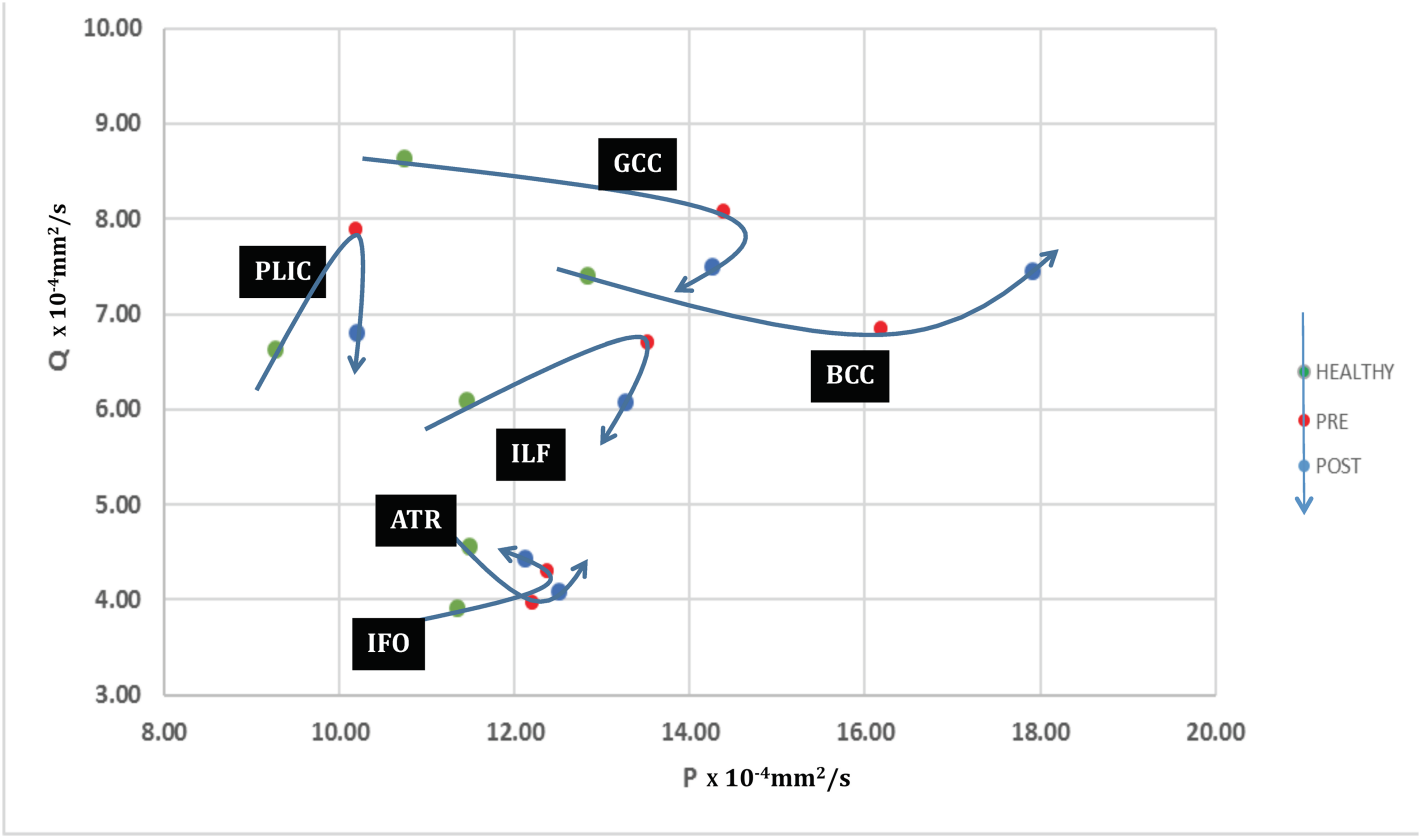
PQ plots of pre-operative and post-operative NPH patients compared to healthy controls. The effect of shunting in NPH patients. Trajectories of six brain ROIs in the p:q plane, illustrating their evolution from control (HEALTHY), to hydrocephalus pre-shunting (PRE) and two weeks after shunting (POST). A round trip would represent that the ROIs have completed a return journey to normal diffusion levels.

In summary, significant differences were found in the DTI profiles of pre-operative patients compared to healthy controls (Fig 2). Mean, axial and radial diffusivities all increased but to different degrees between tracts and with varying contributions to FA. White matter immediately adjacent to the ventricles (corpus callosum) had the greatest increase in radial diffusivity and reduction in FA. The greatest increase in axial diffusivity was in the posterior limb of the internal capsule. Analysis using p:q methodology confirmed that there was an increase in isotropy (p) in all white matter tracts, the magnitude of which depended on the distance from the ventricles; the corpus callosum demonstrated the biggest change in p compared to the posterior limb of the internal capsule, which demonstrated the least increase (Fig 3). There was an increase in anisotropy (q) in the posterior limb of the internal capsule, inferior longitudinal fasciculus and inferior fronto-occipital/ uncinate fasciculi, but a decrease in anisotropy in the corpus callosum and the anterior thalamic radiation.

Red shading designates percentage differences (%) between pre-operative NPH patients vs. healthy controls. Blue shading designates percentage differences (%) between post-operative NPH patients vs. healthy controls.

Interpretation of pre-operative white matter injury patterns—i) Genu of the corpus callosum- the increase in radial diffusivity (L2and3) is > 4 times that of axial diffusivity (L1), although both the L1 and MD are increased. This is consistent with “predominant transependymal diffusion with stretch/compression”; profiles for the body of the corpus callosum were similar, ii) Inferior longitudinal fasciculus—there is an increase in L1 but this change is proportionally lower than the increases in MD and radial diffusivity (L2and3, 1.5 times predominant). This is consistent with “stretch/oedema”; and iii) Posterior limb of the internal capsule—the increase in L1 is predominant (2.8 times that of MD, modest increase in radial diffusivity) and there is no reduction of FA. This is consistent with “predominant stretch/compression”. Using the same methodology, the inferior fronto-occipital/uncinate fasciculi demonstrated “stretch/oedema’ but the anterior thalamic radiation had oedema without stretch (no increase in L1). The overlay of post-operative and pre-operative DTI profiles illustrate changes in NPH patients vs. healthy controls at two weeks post-intervention. Significant changes were demonstrated in the posterior limb of the internal capsule (FA and L1).

#### Early changes post-surgical intervention (Tables 3 and 4)

Clinically, all patients in the study returned to baseline at two weeks post-intervention with no significant post-operative complications. Patients underwent routine post-operative functional optimization including clinical scanning and shunt valve adjustment; there were no significant changes in gait measures or outcomes at this stage, as confirmed by expectations from recommended guidelines [16]. DTI profiles in NPH patients still differed post-operatively from healthy controls (Fig 2). There was no significant change in mean diffusivity in any of the tracts examined. Significant reductions were seen in both axial diffusivity (-6.7%; *p = 0*.*022*) and FA (-9.2%; *p = 0*.*010*) in the posterior limb of the internal capsule, together with a trend towards an increase in radial diffusivity (+35.0%; p = 0.213). The post-operative FA was now less than healthy controls. The only other significant change was an increase in axial diffusivity in the body of the corpus callosum (+7.7%; *p = 0*.*027*). On p:q examination, there were sharp decreases in anisotropy (q) in the genu of the corpus callosum, inferior longitudinal fasciculus and the posterior limb of the internal capsule (Fig 3). Changes seen in the inferior fronto-occipital/ uncinate fasciculi and anterior thalamic radiation were small. Both isotropy and anisotropy increased in the body of the corpus callosum. Overall, only the trajectories of the posterior limb of the internal capsule and inferior longitudinal fasciculus suggested attempts at ‘round trips’, i.e. a return to normality.

## Discussion

### DTI findings of pre-operative NPH patients compared to healthy controls and changes at two weeks following shunting

Our study has shown that, compared to healthy controls, NPH patients exhibited distinct profiles of white matter injury. These profiles can be entirely described by changes in anisotropic indices that are specific to the individual tracts. The most consistent finding, in the six white matter tracts examined in NPH, was widespread increases in mean diffusivity. Superimposed on this general increase in white matter mean diffusivity are tract-specific changes that appear to correlate with both the proximity of the tracts to the lateral ventricles as well as to their directionality. For consistency, we interpreted the white matter injury patterns in this study both in the context of established DTI findings of ‘pure’ models of white matter damage as well as known imaging findings in hydrocephalus (Table 5).

**Table 5:** Pathophysiological basis for DTI interpretation in NPH (see Discussion for references; eq denotes equivocal findings in literature).

Pathological conditions	FA	MD	L1	L2and3	Interpretation	Pathophysiology and relevance to NPH
A broad range of neurological conditions	Low	eq	eq	eq	Reductions in FA are more common; increases in FA are more rarely observed	FA is dependent on the magnitude of changes in axial and radial diffusivities
Aging, leukoaraiosis	eq	High	eq	eq	Small increase in periventricular MD in normal ageing	Age-matched healthy controls are important for the study of NPH
Demyelination	eq	eq	eq	High	Myelin increases the orderliness of white matter fibre tracts	Increased orderliness of tracts is characterized by increased axial, reduced radial diffusivities
Remyelination	eq	eq	High	Low		
Axonal loss	Low	High	Low	High	Changes in FA, MD and axial are features of axonal loss	These changes are likely to be irreversible, demyelination increases radial diffusivity
Vasogenic oedema	Low	High	eq	eq	Pure changes in vasogenic oedema do not result in significant differences in diffusivities	Oedema produces changes sufficient to impact upon FA and MD
Carpal tunnel syndrome, cervical spondylotic myelopathy	Low	High	Low	High	Pure compression in fixed compartments produces a situation without the possibility of stretch	Compression without stretch conversely increases tract tortuosity due to impaction
Displacement around tumours	High	eq	High	Low	Distortion due to space-occupying lesions	Stretch without compression, no impact on MD
Obstructive, paediatric hydrocephalus	eq	High	High	eq	Restricted to periventricular tissues, tend to resolve after CSF diversion	Probably the result of reversal of CSF flow through the ependymal lining
NPH in published work	High Low	High	High	eq	MD histogram analysis able to distinguish NPH from neurodegenerative disorders including Alzheimer’s and Parkinson’s diseases with 86% sensitivity and 96% specificity	MD alone is inadequate to characterize complex post-operative changes; reduction has been demonstrated with external lumbar drainage, yet MD remained significantly different in patients vs. controls two weeks following shunting.
NPH in this study (including propoportions of change)– 1. Predominant transependymal diffusion with stretch/compression	Very Low	Very High	High	Dominant	Loss of integrity with significant compression and oedema sufficient to cause axonal disruption (GCC and BCC; adjacent to ventricles)	Different patterns coexisting, dependent on proximity to the ventricles and orientation of white matter fibres within the tracts. Multifactorial probably including axonal degeneration, demyelination, small vessel disease, enlarged Virchow-Robin spaces, reversal of interstitial fluid flow, increased transependymal CSF diffusion and impaired cerebrovascular autoregulation within the white matter. The component of stretch/ compression, characterized by increased axial diffusivity and anisotropy, appears to be the most amenable to surgical intervention.
2. Stretch/oedema	Low	High	High	High	Loss of integrity with significant compression and oedema (ILF; lateral to ventricles)	
3. Predominant stretch/compression	High	High	Dominant	Moderate	Loss of integrity with predominant compression (PLIC; remote to ventricles)	

In addition, we further performed p:q analysis on the DTI measures; by decomposing them into their quantitative scalar components, it was possible to generate plots of isotropy (p) vs. anisotropy (q), that were not dependent upon prior knowledge of those profiles. When p, q coordinates were plotted in dimensionally comparable graphs, we found that differences in DTI profiles produced patterns of injury that occupied geographically distinct white matter districts (Fig 3). Furthermore, individual ROIs demonstrated differing degrees of early responsiveness to surgical intervention, suggesting that some patterns of white matter injury were more reversible than others.

### DTI findings in demyelination, degeneration, oedema, compression and displacement of white matter

How are DTI findings, consistent with measures described in our study, interpreted within the wider context of white matter injury? Table 5 presents changes seen in DTI measures observed across pathologies. To summarize, reductions in FA are reported in a broad range of conditions whereas increases in FA are more rarely observed. Increases in MD are consistent with conditions involving axonal loss, vasogenic oedema or leukoaraiosis [39–44]. Increases in radial diffusivity occur in demyelination [39, 45]. Compression in fixed compartments, such as carpal tunnel syndrome [46, 47] and cervical spondylotic myelopathy [48, 49] reduces FA, increases MD, reduces axial diffusivity but increases radial diffusivity. Conversely, displacement around tumours results in increased FA, unchanged MD, increased axial diffusivity and decreased radial diffusivity [50]. In NPH, we found that differing and more complex patterns could be observed as compared to DTI changes demonstrated in these purer neurological entities.

### DTI interpretation in the context of hydrocephalus in general

Our demonstration of increased mean diffusivity in all six tracts examined reflects increases in both axial and radial diffusivities and confirms earlier studies. Gideon *et al*. originally reported findings of a generalised increase in brain ADC in five patients with NPH whereas Ivkovic *et al*. have suggested that MRI mean diffusivity histogram analysis is able to distinguish NPH from neurodegenerative disorders including Alzheimer’s and Parkinson’s diseases with 86% sensitivity and 96% specificity [12, 51]. We have shown that this increase in mean diffusivity reflects increases in both axial and radial diffusivities.

With normal ageing, there is a small increase in periventricular mean diffusivity [52]. Increases in mean diffusivity in obstructive hydrocephalus, at least in younger age-groups, are restricted to the periventricular tissues as the result of reversal of CSF flow through the ependymal lining and tend to resolve after CSF diversion [11, 53–55]. By contrast, in NPH, progressive ventriculomegaly occurs in the apparent absence of raised intracranial pressure, yet mean diffusivity changes are more widespread. The mechanism of the more generalized increase in white matter mean diffusivity in NPH is unclear but probably multifactorial, including axonal (Wallerian) degeneration, demyelination, small vessel disease, enlarged Virchow-Robin spaces, reversal of interstitial fluid flow, impairment of cerebrovascular autoregulation within the white matter, and increased transependymal diffusion of CSF. Interestingly, an increase in extracellular water *per se* may have no effect on neurological function unless it contains neurotoxic compounds [56].

### Challenges of imaging theories and mechanisms in NPH

It is clear that NPH is likely to be a balance of multiple injury elements co-existing. Neuroradiological findings have been mechanistically diverse, spanning changes from cerebral blood flow and CSF hydrodynamics to the architecture of structural compartments and gray matter volume [57]. Whilst DTI findings in both purer neurological conditions and acute hydrocephalus are not entirely applicable to NPH in its complexity, there has been evidence in the literature of changes consistent with reproducible injury patterns, particularly in the corpus callosum and posterior limb of the internal capsule, despite the wide variability of technical specifications employed [58]. Yet, not all white matter injury is necessarily amenable to intervention. This limits potential candidate mechanisms with the ability to achieve the concept of plausible reversibility.

One proposed mechanism with apparent merit is stretch. Assaf *et al*. suggested that, in children with acute hydrocephalus, stretching of the fibres to become more linearly aligned results in fewer obstacles to diffusion [11]. Work in NPH by Hattori *et al*. demonstrates their concept elegantly [14]. This is also consistent with findings in MR elastography, that have revealed that brain tissue is softer (reduced shear elasticity) in NPH, accompanied by a decrease in microstructural connectivity [59]. Microstructural connectivity is the only parameter found to improve after shunting [8]. Why should axonal stretch result in enhanced diffusion along the direction of the axons—surely stretch should decrease the extracellular fluid space between axons and thereby restrict diffusion? Conversely, there may also be a limit to axonal stretch beyond which microstructural gaps appear; distortion may turn into ‘leakiness’ resulting in increased diffusion. The concept of stretch alone is clearly insufficient to describe DTI changes found within NPH. Excess extracellular water, another candidate for plausible reversibility, would be consistent with the finding of increased mean diffusivity in this and other studies. However, on its own, oedema would also be insufficient to describe DTI changes found within NPH in their entirety. Stretch does not necessarily occur in other conditions with excess extracellular water; oedema as a sole pathological process would be contradictory to some of the DTI findings in this study. Our approach to resolving such conflict is by describing the finding of stretch in its proper context. In cases where stretch increases the linearity of axonal fibres and results in increased axial diffusivity, we prefer the term ‘*stretch/ compression’*. Whilst stretch remains the key concept, the combination term acknowledges the contradictory possibility of decreases vs. increases of radial diffusivity, such as seen in distortion around brain tumours vs. compression in fixed neural compartments. In the context of severe distortional stretch of fibres resulting in a larger component of increased radial diffusivity, we prefer the term ‘stretch/oedema’, which conveys the concept of leakiness across axons where stretch has been sufficient to result in compromised microstructure.

In imaging NPH, there is a difficult dichotomy between the apparent simplicity of features required for its diagnosis and the challenge of finding adjuncts proven to increase diagnostic accuracy of shunt-responsive NPH. Structural sequences are key to identifying significant ventriculomegaly, not entirely attributable to cerebral atrophy or congenital enlargement, and for confirming that there is no macroscopic obstruction to CSF flow [17]. However, in NPH, many other features contribute in small but meaningful ways to understanding shunt-responsiveness, such as periventricular lucency, degree of leukoaraiosis, hyperdynamic CSF flow, diameter of the corpus callosum, calculation of the callosal angle, tightness of the high convexity, perihippocampal and subarachnoid space morphology as well as changes in cerebral blood flow and perfusion [2, 5, 17, 57, 60–67]. It is difficult to reconcile the need for additional advanced correlates of disease vs. practicalities, such as time constraints, of scanning patients with dementia. Non-invasive imaging that can be acquired rapidly and methodology deliverable at lower specifications, such as part of routine clinical imaging, would greatly enhance our ability to screen for patterns potentially amenable to intervention. NPH presents as a spectrum; responsiveness of CSF shunting is imperfect as is prognostication of response. Interpretation of injury patterns cannot be readily solved by global methods alone. What would be the pathophysiological basis of white matter changes that represent a reversible pattern of injury but lack a specific histological signature? DTI is a tool capable of revealing damage to microarchitecture of axons without presupposing knowledge of the underlying mechanism of such injury. The major obstacle to a more immediate application of DTI in NPH is a transparent and reproducible method of interpretation that is both achievable within a clinical setting and yet takes into account such critical gaps in knowledge.

### DTI—From tensor ellipses to profiles

In this study, we established a basis for the interpretation of DTI changes in NPH based on set patterns involving purer pathologies, *a priori*, in order to resolve such conflicts (Table 5). By examining each ROI set for their full panel of DTI measures simultaneously, we were able to generate DTI profiles that represent both directional information, as well as proportional magnitude. It was possible to appreciate predominate patterns of injury and illustrate them in the form of radar graphs that are scalar and dimensionally comparable (Fig 2).

An increase in MD and reduction in anisotropy are shared by many disease processes [68]. Examination of FA and MD alone are insufficient to fully characterize patterns of white matter injury. It has been well described that directions of change may be underestimated by conflicting changes in individual measures [35, 69]. The interpretation of FA requires knowledge of both axial and radial diffusivity components [69]. This concept has already been established in DTI analysis, for example, the patterns of compression differ depending on context, such as displaced/ stretched fibre systems in brain tumours [50] vs. cervical spondylotic myelopathy. Simultaneous changes in both components may dilute the overall direction of change in FA. FA is mainly dependent on the change in axial diffusivity, i.e., its larger component. Modest but significant changes in radial diffusivity, its smaller component, may not impact on FA as greatly as small changes in its larger component. Conversely, a large change in radial diffusivity may unduly mask the contribution of modest changes in axial diffusivity. These considerations are well known in DTI interpretation, yet do not greatly impact the analysis of findings in conditions such as tumours or stroke, for which histological findings are described. As NPH is a disease that lacks a histopathological signature, such correlations are lacking. Corroboration of DTI findings depends on other methods, such as multi-modal imaging and responsiveness to CSF drainage. Our methodology of DTI profiling and correlation with PQ graphs, whilst seeming more laborious when compared to semi-automated methods, has the advantage of demanding consistency within DTI measures, in order to avoid such pitfalls in interpretation.

### Patterns of white matter injury in NPH pre-operatively and early changes following surgical intervention

DTI profiles revealed four main patterns of changes (three pre-operative and one post-operative) that appeared to be influenced by neuroanatomical factors. We found that both the proximity of white matter tracts to the lateral ventricles and the directional arrangement of fibre tract bundles mattered. In general terms, tracts directly adjacent to the ventricles, such as the corpus callosum, were the most affected, with significantly abnormal findings across the entire panel of DTI measures. Tracts furthest away from the ventricles, arranged in a supero-inferior fashion, such as the posterior limb of the internal capsule, were the most preserved. Fibre tracts lateral to the ventricles, running antero-posterior, demonstrated a combination of the injury elements observed in the other two patterns.

### Pre-operative changes in NPH patients compared to healthy volunteers

#### i) Predominant transependymal diffusion with the presence of stretch/compression

The most affected white matter tracts were the genu and body of the corpus callosum that are directly adjacent to the ventricles, consistent with other studies [70, 71]. The decreases in FA and anisotropy (p, q components) were due to a large increase in radial diffusivity despite smaller increases in axial diffusivity (Figs 2 and 3). By comparison with the DTI changes of white matter injury seen in more straightforward diseases (demyelination, infarction, vasogenic oedema, leukoaraiosis, carpal tunnel syndrome, cervical myelopathy and displacement around tumours) [39–50], the increase in MD and loss of anisotropy in the tracts adjacent to the ventricles (corpus callosum) are compatible with axonal disruption (Wallerian degeneration) and reversal of CSF flow through the ependyma. This results in expansion of the extracellular space (interstitial oedema). As oedema alone would not cause increases in axial diffusivity, a smaller component of stretch/compression must exist. However, the proportional magnitude of decreased FA and increased MD strongly favour oedema as the predominant process, here specifically termed transependymal diffusion due to its location.

#### ii) Stretch/oedema

The tracts lateral to but close to the ventricles, inferior longitudinal fasciculus, demonstrated similar but less severe changes consistent with stretch/compression and increased interstitial fluid. DTI findings in the inferior fronto-occipital/ uncinate fasciculi were also consistent with this profile, despite being further away from the ventricles. There is some, albeit limited, neuropathological evidence to support these interpretations [6, 72]. The impact of proximity to the ventricle may be a reflection of greater tissue distortion not only on adjacent axons but also on periventricular vasculature, leading to increase in local cerebrovascular resistance and ischaemia, with resultant watershed infarction and impairment of regional cerebrovascular autoregulation [73–75]. The anterior thalamic radiation demonstrated moderate increase in fluid, i.e. oedema, but without stretch. In this pattern, significantly increased axial diffusivity, the hallmark of compression, was lacking. DTI findings favoured increased diffusion of fluid through compromised microstructure of axons, with or without the finding of distortional stretch.

#### iii) Predominant stretch/compression

By contrast, the posterior limb of the internal capsule demonstrated the greatest increase in axial diffusivity and anisotropy (p, q components), a modest increase in MD and radial diffusivity with no reduction in FA (Figs 2 and 3). These changes in the posterior limb of the internal capsule confirm previous studies [14]. Findings are consistent with predominant displacement/stretch and a modest increase in interstitial fluid; none of the changes are sufficiently severe enough as to impact on overall FA. This pattern is the closest finding in NPH that mirrors the increases in axial diffusivity have been shown to accompany acute hydrocephalic conditions [11]. However, differences still remain; decreases in radial diffusivity have been shown to accompany acute hydrocephalus [11]. This is probably reflective of the purer nature of the condition, in which the stretch pattern of linearly aligned axons with reduced extracellular fluid spaces between them occurs due to raised intracranial pressure, a feature absent in NPH.

### Early post-operative changes at two weeks

At two weeks following surgery, it was possible to demonstrate significant changes in axial diffusivity in the posterior limb of the internal capsule. There was also a modest increase in radial diffusivity, consistent with re-expansion of extracellular fluid spaces between axons. These changes are consistent with relaxation of stretch/compression following surgical intervention. The findings preceded changes in clinical outcome; whilst the significance of such results requires further clinical correlation, they nevertheless provide evidence of changes in DTI directly attributable to the effect of surgical intervention upon white matter injury patterns. Surgery did not impact upon all three pre-operative injury patterns in the same way; the pattern of predominant stretch/ compression was the most amenable to intervention. However, trends demonstrated such DTI changes following intervention could be seen for the profile of stretch/compression even if it was not predominant; changes were proportional to the magnitude of the component present pre-operatively. Conversely, other profiles progressed despite surgical intervention, implying that the absence of the profile of stretch/ compression may be disadvantageous, or indeed that other profiles of white matter injury are less reversible.

### Correlation of DTI profiles to p:q tensor decomposition methodology

We further correlated the changes seen using DTI profiles with p:q methodology [35]. As described above, this visualization method, based on tensor transformation, decomposes the diffusion tensor into its isotropic (p) and anisotropic (q) components. The advantage of this technique is the possibility of simultaneous analyses of both FA and MD, as well as an additional five scalar measures. Changes in isotropy and anisotropy were concurrently graphed in two equivalent axes to allow them to be dimensionally comparable. This allows of changes to be represented without *a priori* presumptions of predominant pathological injury. We found an increase in the isotropy of all white matter tracts as all ROIs evolved from healthy controls to pre-operative NPH patients, consistent with the interpretation of widespread axonal disruption or distortion (Fig 3). As demonstrated using DTI profiles, the degree of increase in MD and isotropy measures was dependent upon the distance from the ventricles. Changes in the corpus callosum were the most severe and indicative of axonal disruption whereas the posterior limb of the internal capsule was relatively preserved, with findings more suggestive of axonal distortion by stretch/compression. This is consistent with work using regional cerebral blood flow, the findings of which have demonstrated more congruence with distance to ventricles than functional neuroanatomical groupings [5, 66]. Anisotropy increases seen on the p:q plane in the posterior limb of the internal capsule, inferior longitudinal fasciculus and inferior fronto-occipital/ uncinate fasciculi supported the interpretation of stretch/compression. In the corpus callosum and the anterior thalamic radiation, changes in isotropy predominated, consistent with increased transependymal diffusion and interstitial fluid i.e. oedema.

Two weeks post-surgical intervention, p:q changes for the genu of the corpus callosum, inferior longitudinal fasciculus and the posterior limb of the internal capsule suggested improvement in stretch/compression (Fig 3). Only two ROIs, the posterior limb of the internal capsule and inferior longitudinal fasciculus, demonstrated trajectories attempting a ***‘round trip’***; i.e. a return journey to normal levels of p:q diffusion characteristics. When the DTI and p:q profiles were compared to stretch curves modelling the relationship between mechanical stretch (L) and diffusion tensor parameters, the post-operative changes seen in the genu of the corpus callosum, inferior longitudinal fasciculus and the posterior limb of the internal capsule were consistent with the theoretical prediction for decompression. Strikingly, changes in DTI and p:q profiles suggested that the body of the corpus callosum apparently worsened. We hypothesize that this may simply be a ‘shunt effect’. Changes in radial diffusivity and isotropy do not distinguish between inflow and outflow movements; in the early post-operative period, it is therefore not possible to distinguish between the continuing pathological process of transependymal diffusion vs. the increased flow into the ventricles due to the physiological process of CSF drainage due to shunting. This is in contrast to finite periods of continuous CSF diversion, such as external lumbar drainage, in which changes of decreased mean diffusivity could be more simply demonstrated [76]. The effects of producing different results in DTI measures in the context of differing conditions of CSF diversion, such as lumbar drainage vs. definitive shunting, reinforce the advantages of using a methodology of DTI profiles correlated to PQ graphs as this offers more consistency in the interpretation of changes across differing flow characteristics.

### Correlation with clinical features of NPH

This study demonstrates the responsiveness of differing DTI profiles of white matter injury to surgical intervention. Demonstrating changes due to intervention does not necessarily equate to demonstrating clinical improvement secondary to shunting. In fact, as is quite commonly the case clinically, patients in this study were seen to merely return to baseline at this stage, following a period of post-operative functional recovery. It is for this reason that post-operative clinical outcome assessment, involving the use of NPH outcome scoring, is usually recommended only at 3-, 6- and 12-months following surgery [16]. Outcome measurements beyond one year are heavily influenced by the presence of comorbidities in the ageing cohort whilst scoring prior to 3-months is confounded by multiple factors, such as the need for post-operative functional optimization, shunt valve setting adjustment and the presence of subdural fluid collections of uncertain significance, amongst other considerations. However, despite such qualifications, the study does demonstrate the effect of surgical intervention on patterns of white matter injury prior to changes in clinical function. Such a baseline is important to establish; published studies have either focused on enhancing tools for the differential diagnosis of NPH vs. neurodegenerative conditions or correlating the effect of shunting with clinical outcome groups. What is the tissue signature compatible with reversible injury and what is the effect of CSF drainage on such patterns? This study seeks to contribute to the understanding gap that exists between the pathophysiological evolution of NPH and the degree of responsiveness of pathological processes to intervention.

Pre-operative DTI profiles offered insights into the differing white matter injury patterns found in NPH. Paradoxically, despite the pre-operative DTI evidence of stretching in the posterior limb of the internal capsule, the gait disturbance of NPH is not accompanied by any signs of a pyramidal disturbance and there are only subtle effects on hand function. Central motor conduction is unaffected in NPH. Despite the widespread DTI changes in the corpus callosum, there is no evidence, except for two case reports, of neuropsychological features of a callosal disconnection syndrome. The cognitive profile of NPH is subcortical in nature; this may be consistent with the subtle white matter injury patterns seen. There are published reports of changes in volume of relevant structures, including the hippocampus and fornix, as well as the caudate nucleus, but the mechanisms of these changes are unclear [77, 78]. Although our results demonstrated congruence with DTI findings from a spectrum of hydrocephalic disorders, the severity and extent of axonal distortion demonstrated by our findings are disproportionate to increases in MD predicted by work in normal aging, paediatric and obstructive hydrocephalus [51, 52, 79]. It appears that the pathophysiology of NPH its distinct from other related conditions.

### Limitations

The study presents a dataset acquired using modest DTI specifications. This is consistent with other published studies in literature demonstrating similar findings [11, 12, 14, 71, 80–82] and reflects the specific challenges of imaging this patient population. Valve implants present further challenges in the post-operative phase, with the blooming artefact associated with magnetic valves possibly worse at higher resolutions. There are also other considerations of scanning at higher specifications; a recent safety advisory has recently been issued against a valve in extensive clinical use, declaring a previously unknown additional safety risk of scanning using the MR-compatible valve, but only at 3-Tesla. As patients with NPH who undergo surgical intervention serve as their own controls, a further consideration is the issue of repeatability of DTI acquisition between imaging sessions. However, data from in-house studies using the same MR system, sequences and radiographic staff in healthy subjects aged 25–44 years have demonstrated that variability in reproducibility data from within and between sessions was lower than the values for inter-subject variability [83]. Standard thresholds for changes in DTI parameters are not yet available despite many methodological studies. Nevertheless, further work is underway to produce reference data using specific predetermined DTI thresholds for future comparisons in NPH patients and age-matched healthy controls.

The degree of ventriculomegaly also differs greatly between patients in NPH, resulting in technical difficulties in generating accurate data from various white matter quantification techniques that rely on computer-generated masks and normalized brain templates, such as Tract-Based Spatial Statistics (TBSS) and Voxel-Based Morphometry (VBM). In addition, the DTI model needs to be sufficiently subtle such that it is possible to observe differences for ROIs selected *a priori* in patients pre- and post-surgical intervention, that would also allow comparisons of patients against themselves as controls. The results indicate that the DTI methodology using ROIs is able to demonstrate white matter injury in NPH patients compared to healthy controls. ROIs were chosen to minimise the well-known problems posed by crossing fibres and CSF contamination [68, 84]. Such manual methods are not without their drawbacks but the advantages of ROI methodology are both its accessibility and clinical familiarity with its use. In this study, we have confirmed that high intra- and inter-rater reliability is possible using strict neuroanatomical landmarks. The participant groups could also be criticized for being small; however, such numbers have proven adequate to demonstrate significant differences between patients (pre-operative and post-operative) and healthy controls. In the case of mean diffusivity, 15 patients were sufficient for sensitivity/specificity analysis [12]. Our findings of responsiveness of white matter injury patterns to intervention need to be confirmed with clinical outcome. Yet, our demonstration of pure DTI changes due to shunting, that preceded changes in clinical symptomatology, forms a useful baseline in assessing the degree of differences required to effect meaningful improvement in patients with this condition.

## Conclusions

Few, if any, neurological disorders in elderly patients have demonstrated improvement to the levels of 60–80% with intervention as reported in NPH [85]. Methods to define a potentially remediable hydrocephalic component in the individual patient remain controversial. Novel, yet minimalistic imaging strategies that are readily translatable to immediate clinical use would be urgent priorities. DTI profiling and p:q correlation to characterize patterns of white matter injury may contribute a non-invasive biomarker of potentially reversible white matter injury and risk-stratification for surgery particularly in patients whose clinical phenotype overlaps with other neurodegenerative diseases. Future work to corroborate patterns with discrete clinical outcome groups, neurodegenerative diseases, other forms of acute-on-chronic hydrocephalus, as well as overlay of significant comorbidities, would be invaluable.
